# Depth of Cure of a Simplified Bulk-Fill Universal Composite and a Conventional Resin-Based Composite

**DOI:** 10.3390/ma19122657

**Published:** 2026-06-20

**Authors:** Alexis Maquère, Darien DeWolf, Daniel Labrie, Richard B. Price

**Affiliations:** 1 Department of Physics and Atmospheric Science, Dalhousie University, Halifax, NS B3H 4R2, Canada; alexis.maquere@2025.icam.fr (A.M.); daniel.labrie@dal.ca (D.L.); 2 Department of Dental Clinical Sciences, Dalhousie University, 5981 University Avenue, Halifax, NS B3H 4R2, Canada; 3 Department of Mathematics and Statistics, St. Francis Xavier University, 4130 University Ave, Antigonish, NS B2G 2W5, Canada; ddewolf@stfx.ca

**Keywords:** Vickers hardness, resin-based composites, photocuring, depth of cure

## Abstract

Objectives: This study evaluated the influence of shade, irradiance, and exposure time on the depth of cure (DoC) of a simplified bulk-fill universal composite (Tetric plus Fill) and a conventional composite (Filtek Supreme Ultra). Methods: The 80% bottom-to-top hardness ratio was used as an empirical cutoff, and 99% confidence intervals were calculated using R version 4.5.1. Results: The DoC values were material and protocol dependent. Tetric plus Fill reached the 80% threshold to depths ranging from 3.5 to 4.5 mm, depending on shade and exposure protocol. All the Tetric plus Fill and the Filtek Supreme Ultra products reached their manufacturer’s claimed DoC depth with both 3 s extra-high and 10 s high exposures from the Bluephase PowerCure. Significance: This study highlights the importance of following the manufacturers’ recommendations for increment thickness and exposure time and of recognizing that shade and material formulation influence DoC.

## 1. Introduction

According to the World Health Organization, dental caries (tooth decay) is the most common non-communicable disease worldwide, affecting over 2.3 billion people [1,2,3]. Successfully restoring these decayed teeth using resin-based composites (RBCs) relies heavily on adequately photopolymerizing the RBC [4,5,6,7]. This RBC polymerization is related to the degree of conversion (DC) of the resin from monomer to polymer and is a critical parameter that influences the depth of cure (DoC) and mechanical properties of the RBC [4,6,7,8,9]. Inadequate polymerization of the RBC can lead to compromised mechanical strength, increased wear, reduced marginal integrity, and potential biological issues due to the release of unreacted monomers. Inadequately photocured RBCs also promote more bacterial growth and are associated with increased *Streptococcus mutans* biofilm formation [10]. Consequently, inadequately photocured RBCs are more prone to causing secondary caries [10]. It is also well reported that inadequately photocured RBCs release more undesirable acrylate monomers into the oral cavity [10,11,12,13,14,15,16,17]. Therefore, it is important to ensure that dental resins are adequately photocured.

The photopolymerization of RBCs is influenced by several factors, including the light-curing unit’s (LCU’s) light output and emission spectrum, exposure time, the shade and opacity of the RBC, and increment thickness [4,7,18]. Manufacturers of conventional RBCs typically recommend photocuring their RBCs in increments that are no more than 1.5 to 2 mm thick to ensure sufficient polymerization throughout the restoration. However, most dentists cannot accurately gauge the thickness of each increment of RBC that they place into a cavity [19]. Bulk-fill RBCs were developed to simplify placement by allowing thicker increments and reducing the number of placement and light-curing steps [20,21]. This simplification is useful only if the material can be adequately polymerized throughout the recommended increment thickness; therefore, it remains important to verify whether bulk-fill RBCs meet the manufacturer’s claimed 4 mm DoC.

The DoC can be determined using the ISO 4049 standard test method [22]. In the ISO 4049 test, the RBC specimen is made in a 4 mm diameter stainless-steel mold over white filter paper. The specimen is removed immediately after light exposure, and the uncured RBC is scraped away immediately using a plastic spatula. The maximum length of the remaining hard RBC is then measured and divided by two. While this ISO 4049 DoC test [22] offers simplicity and ease of implementation, operator technique [23], the type of mold material, backing, and mold diameter [24,25,26,27,28,29], as well as the beam profile from the LCU [30,31], are known to affect the DoC values obtained using this test. In 2019, it was reported that the 4 mm diameter metal mold used in the ISO 4049 test was a conservative method for determining the DoC of RBCs [29]. The use of a 6 mm diameter metal mold is closer in diameter to a tooth than a 4 mm diameter mold. Using a 6 mm diameter mold has been reported to increase the DoC by 8% [27], and several studies have used a 6 mm diameter metal mold [32,33] to better represent the size of a posterior cavity.

Instead of scraping away the uncured RBC, Vickers microhardness testing has been used as an alternative method for assessing the DoC of dental RBCs [9,34,35,36], and microhardness values have been reported to correlate with the degree of monomer conversion of the specific RBC being tested [37,38]. By measuring the microhardness of the RBC at different depths and comparing it with the top surface hardness, the DoC can be determined. This DoC value is often defined as a percentage of the maximum surface hardness, and if the RBC achieves a bottom-to-top hardness ratio of at least 80%, the RBC is usually considered to be adequately polymerized [9,36,39,40,41]. This 80% ratio is an empirical screening criterion rather than a precise biological threshold; a value just below 80% does not necessarily indicate immediate clinical failure, but reduced hardness at depth is generally associated with lower polymerization, which may be accompanied by reduced mechanical properties, lower elastic modulus, and greater elution of residual monomers [4,5,6,7,8,9,10,11,12,13,14,15,16,17].

Therefore, this study used the Vickers microhardness test to investigate the influence of two light exposure times and irradiance levels from the same LCU on the depth of cure of a simplified bulk-fill universal RBC, Tetric plus Fill (Ivoclar, Schaan, Liechtenstein) [42], in all four available shades when compared to a conventional RBC, Filtek Supreme Ultra (Solventum, St. Paul, MN, USA), which is known to have acceptable clinical performance [43,44,45], but is expected to achieve only a 2 mm depth of cure [46]. The RBCs were exposed for either 3 s in the 3 s extra-high irradiance mode or 10 s in the high irradiance mode, using the same LCU under identical conditions. The null hypotheses were:

1. The four shades of Tetric plus Fill would not reach the manufacturer’s claims of a 4 mm depth of cure after a 10 s exposure using the Bluephase PowerCure [42].
2. For the tested RBCs, the 3 s extra-high irradiance mode would not achieve the same depth of cure as the 10 s high mode using the Bluephase PowerCure.
3. Filtek Supreme Ultra would not reach the manufacturer’s claims of a 2 mm depth of cure in 3 s using the extra-high irradiance mode of the Bluephase PowerCure [46].

## 2. Materials and Methods

### 2.1. Materials

This study examined all four shades (A2 plus, Bleach plus, A3 plus, and A3.5 plus) of Tetric plus Fill (Ivoclar). This is a simplified bulk-fill universal RBC, specifically designed for photopolymerization in a single increment up to 4 mm thick. For comparison, one conventional RBC, Filtek Supreme Ultra A2B, was included as a control because it is manufactured to be photopolymerized in increments that are no more than 2 mm thick. The composition of the RBCs, as provided by the manufacturers, is reported in Table 1.

### 2.2. Sample Preparation

The RBC specimens were prepared in cylindrical stainless-steel molds with an internal diameter of 6 mm and a depth of 10 mm. Before each use, the mold was cleaned using ethyl alcohol and dried. The RBC was then carefully inserted into each half of the cylindrical mold under filtered orange light to prevent premature curing of the RBC. To separate the RBC in each half of the mold and ensure a uniform flat surface, the uncured RBC was covered with a 25 µm thick optically clear polyester film (48-1F-OC-100, CS Hyde Company, Lake Villa, IL, USA) and the RBC in each half of the mold was then pressed flat using a glass slab. The two flat halves of the semi-cylindrical mold were then assembled face-to-face to form a cylinder of RBC that was separated by the two 25 µm thick polyester strips covering the RBC in each half of the mold. The mold was then placed on a washer with a 12 mm internal diameter hole. A polyester sheet was placed on top to press the top surface flat, and the excess RBC was removed from the bottom of the 10 mm-deep mold with a sharp blade.

**Figure 1:**
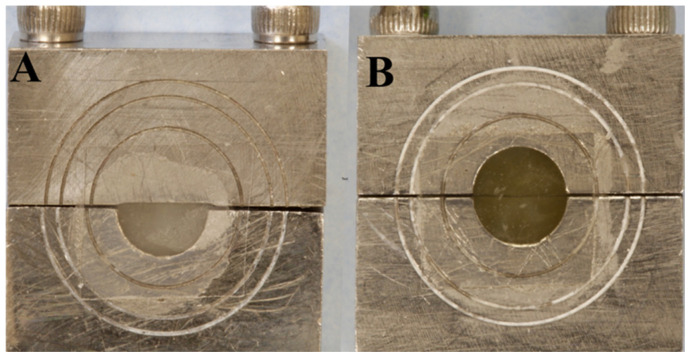
(**A**) RBC filling just one side of the stainless-steel half-cylindrical mold with the RBC pressed flat against the metal. (**B**) A 25 µm thick polyester sheet was placed over the RBC in each half of the mold, and the two halves were then joined to form the cylindrical mold. The scribed circles were used to center the light tip over the RBC.

During a pilot test, six samples of each mold configuration (half-cylinder against metal [Figure 1A] and split cylinder [Figure 1B]) were made. After storing the RBCs for 24 h, microhardness measurements were taken at depths of 0.5 to 5 mm in 0.5 mm increments, at the center of each RBC. Three measurements were made at each depth. An average hardness-versus-depth plot showed that the RBCs made in both mold types achieved approximately 80% of the top hardness at the 4 mm depth. However, the cylindrical mold design, with each half separated by a 25 µm-thick polyester sheet, as illustrated in Figure 1B, was chosen because the microhardness was greater in this full-cylinder configuration. The half-cylinder mold configuration (Figure 1A), where the RBC was adjacent to the metal wall, produced lower hardness values.

### 2.3. Photopolymerization Protocols

The RBC specimens were photocured using the same Bluephase PowerCure LCU (Ivoclar, Schaan, Liechtenstein; serial number 1428007901). The LCU was used with the manufacturer’s 10 > 9 mm diameter light guide. The tip diameter was larger than the 6 mm diameter mold and therefore completely covered the RBC test specimen during exposure.

**Figure 2:**
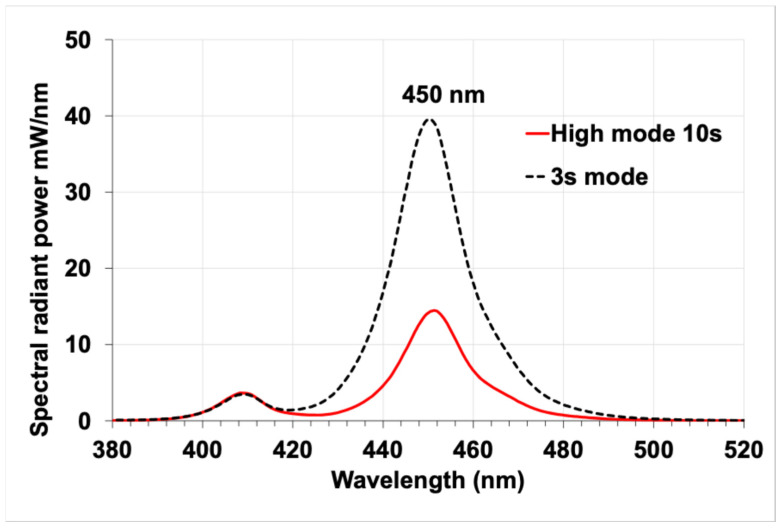
Emission spectrum of the PowerCure measured through a 6 mm aperture at 0 mm distance in the 10 s High irradiance and the 3 s extra-high irradiance modes.

The light output from the LCU was measured in triplicate using a 15 cm diameter integrating sphere connected to a fiber-optic spectrometer (USB 4000, Ocean Optics, Orlando, FL, USA). The measuring system was calibrated before the experiment using a light source traceable to a National Institute of Standards and Technology standard. The light emission at the tip of the light guide was also evaluated by recording the spectral radiant power three times through a 6 mm diameter aperture into the same integrating sphere. This aperture was the same diameter as the diameter of the 6 mm mold. Figure 2 shows the emission spectrum of the PowerCure measured through the 6 mm aperture.

Two different light exposure protocols were used: a 3 s exposure delivering an extra-high irradiance of 3.37 W/cm^2^ using the 3 s extra-high irradiance mode, and a 10 s exposure delivering an irradiance of 1.27 W/cm^2^ using the high mode [47]. When photocuring, the LCU tip was centered directly over and in direct contact with the polyester film covering the surface of the RBC. All sample preparation and photocuring were conducted at a controlled room temperature of 23 °C in an environment free from wavelengths of light below 500 nm.

### 2.4. Vickers Microhardness

After photocuring, the samples were stored for 24 h at 37 °C in the dark and exposed to air to allow most of the post-curing of the RBC to occur [48,49,50].

**Figure 3:**
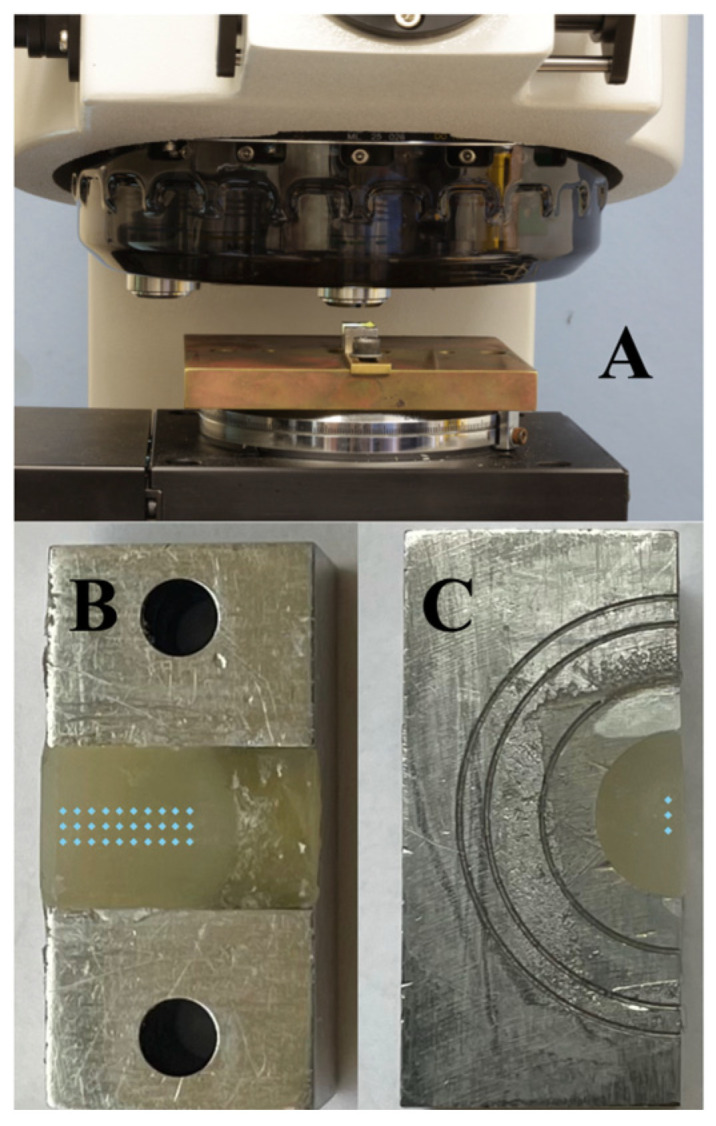
The Mitutoyo HM-220D hardness tester (**A**). The points in (**B**,**C**) indicate where the microhardness measurements were made down the side (**B**) and at the top (**C**) of the RBCs. Note in (**B**) the uncured RBC at the bottom furthest from the top, resulting in a ‘bullet-shaped’ appearance of the cured RBC.

**Figure 4:**
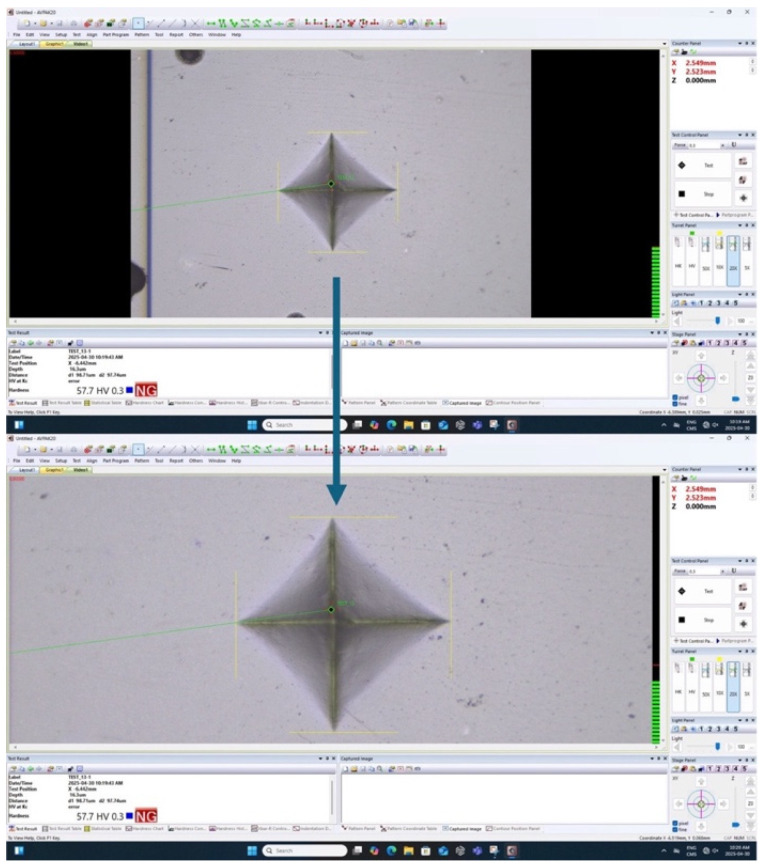
Screenshot of the Mitutoyo AVpak Version 3.1 software showing the image enlarged on the computer screen when measuring the Vickers hardness.

The Vickers microhardness (HV) was measured at the surface and at each 0.5 mm increment down the side of the composites using a Mitutoyo HM-220D micro-Vickers tester (Mitutoyo Canada, Mississauga, ON, Canada). The RBCs were not polished [51,52] because polishing could have disproportionately increased the DoC values at the undercured bottom compared with the well-cured top surface [53]. The Vickers measurements were made using a 300 g indenter load for 8 s. A map of where hardness was measured is shown in Figure 3. As depicted in Figure 4, the resulting image was magnified to fill the computer screen using optical and electronic zoom before it was measured. The Vickers microhardness was calculated from the resulting indentation using the Mitutoyo AVpak Version 3.1 software.

Preliminary investigations into the use of Knoop or Vickers hardness showed that Knoop indentations were more prone to elastic recovery after unloading because the Knoop indentation is highly asymmetric when the RBC is less well cured. The load was selected by examining indentations produced using various loads. It was determined that a 2 kg load could potentially dislodge the RBC from the metal mold or cause surface damage to the photocured RBC, whereas a 50 g load was too low and produced inconsistent measurements. Based on this preliminary study and the loads used in other studies [52,54,55,56,57], an optimal testing load of 300 g was selected to ensure accurate and reproducible Vickers microhardness measurements.

Although the Mitutoyo AVpak software allowed the microhardness indentations to be made automatically, pilot testing showed that the measurements made by a trained user were more reliable. The Vickers microhardness measurements were taken at the top surface and at 10 depths below the surface, from 0.5 to 5.0 mm in 0.5 mm increments. All microhardness measurements were taken in triplicate, including those made at the center of the top surface.

### 2.5. Sample Size and Total Measurements

Based on a power analysis, a total of seven specimens (n = 7) were made for each material and exposure time combination. With five RBC materials (four Tetric plus Fill shades and one Filtek Supreme Ultra) and two exposure times (3 s and 10 s), this resulted in 5 materials × 2 exposure times × 7 repetitions = 70 test specimens. Specimen preparation and testing order were randomized across material and exposure groups. The specimens were coded before hardness testing to blind the examiner to the RBC and photocuring protocols during the hardness measurement. Specimens with visible voids on the measurement surface, incomplete mold filling, detachment from the mold, or gross surface damage were considered defective and were remade before testing. For each specimen, three HV measurements were made at each of the 11 locations for a total of 33 HV data points per specimen. As a result, 2310 HV data points were recorded for this study. The designation “too soft” was used when the material at a given depth was insufficiently polymerized to support a measurable Vickers indentation with clearly defined diagonals using the 300 g load; these locations were not assigned numerical HV values and were not used to define DoC.

### 2.6. Depth of Cure

The Depth of Cure (DoC) was assessed based on a bottom-to-top hardness ratio of ≥80% [9,36,39,40,41]. The Vickers microhardness values were summarized by computing the mean and associated 99% confidence interval (CI) at each depth (0.0 to 5.0 mm in 0.5 mm increments), separately for each RBC and curing protocol (3 s or 10 s). For each group (RBC × protocol × depth), the sample mean and standard error were calculated from replicate measurements, and 99% confidence intervals were constructed. Third-order polynomials were fitted to the data to provide a visual guide only; the polynomial fits were not used to extrapolate the DoC beyond the measured 0.5 mm depth intervals.

### 2.7. Statistics

To quantify curing performance, an 80% bottom-to-top hardness cutoff was defined separately for each RBC by pooling the top-surface hardness values from both exposure protocols for that RBC; thus, the cutoff was material-specific and was not pooled across different RBCs. Each top-surface hardness value at 0.0 mm was multiplied by 0.80, and the resulting set was treated as a derived distribution of 80% thresholds. The mean of these 80% values was used as the cutoff reference, and its 99% CI was visualized as a shaded or thickened band in the graphical plots. The RBC was deemed to fail to meet manufacturer specifications at the depth where the 99% confidence intervals for that RBC were completely below the 99% confidence DoC band. All analyses were conducted in R (version 4.5.1) [58].

## 3. Results

**Figure 5:**
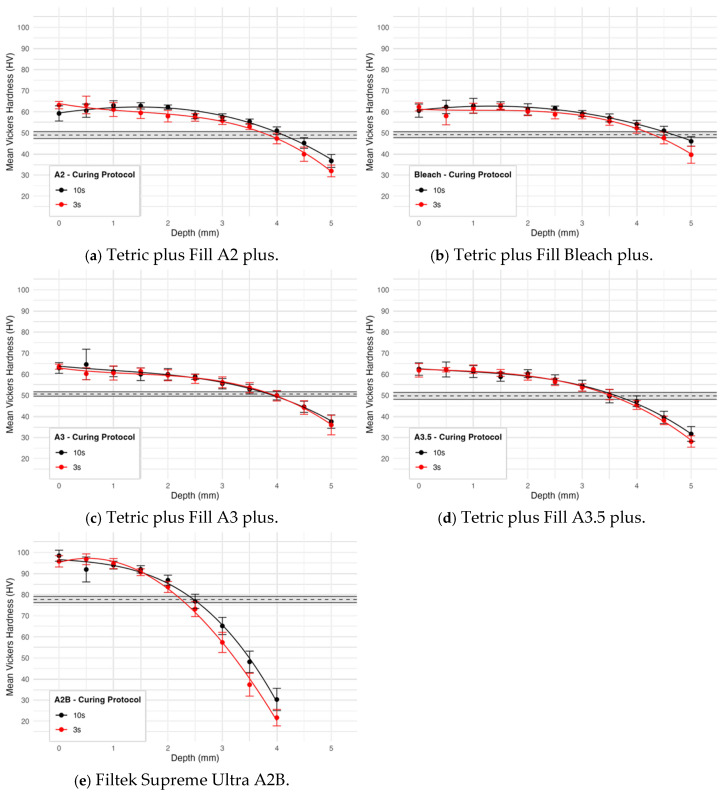
(**a**–**e**) Mean Vickers microhardness (HV) as a function of depth for each composite (RBC) and exposure protocol. Data points indicate observed means, with vertical bars representing 99% confidence intervals. Solid curves generated using cubic polynomial fits to the observed means provide a visual guide. The grey dashed horizontal line denotes the mean 80% hardness cutoff for each RBC, and the thin solid lines above and below the dashed horizontal line mark the upper and lower confidence limits, so that the bar thickness is proportional to the 99% confidence interval of the cutoff.

In the 3 s extra-high irradiance mode, the power through the 6 mm aperture was 952 mW (irradiance 3.37 W/cm^2^). In the 10 s high mode, the power was 359 mW (irradiance 1.27 W/cm^2^). Thus, in the 3 s mode, the radiant exposure was 10.1 J/cm^2^, and in the 10 s mode, it was 12.7 J/cm^2^. The mean Vickers microhardness (HV) values for each RBC as a function of depth and curing protocol are summarized in Table 2 and Figure 5. The 80% bottom-to-top value and standard error (SE) are indicated for every RBC. For all RBCs, surface microhardness (0.0 mm) was highest, with a progressive decline observed as depth increased from 0.0 to 5.0 mm.

The 0.0 mm HV values are reported in Table 2 because they provide the top-surface reference for calculating the 80% bottom-to-top ratio. No statistical comparison of surface hardness among the RBCs was performed, and these surface values should not be interpreted as a separate outcome beyond their role in defining the DoC cutoff.

Figure 5a–e show the mean HV values for the 3 s and 10 s curing protocols within each RBC group. The 80% surface hardness cutoffs, calculated as the mean of the distribution of 80% of surface HV for each RBC, were: Filtek Supreme Ultra A2B: 77.69 (SE 0.55); Tetric plus Fill A2 plus: 48.95 (SE 0.61); Tetric plus Fill A3 plus: 50.60 (SE 0.39); Tetric plus Fill A3.5 plus: 49.71 (SE 0.61); and Tetric plus Fill Bleach plus: 49.14 (SE 0.52). These thresholds are shown as horizontal bands in Figure 5a–e, with thickness proportional to their respective 99% confidence intervals. Figure 5 shows that none of the Tetric plus Fill RBCs or the Filtek Supreme Ultra A2B had 99% confidence intervals that were completely below the 99% confidence DoC band at their advertised DoC.

## 4. Discussion

This study used Vickers microhardness to determine the depth of cure for all four shades of a recently introduced simplified bulk-fill universal composite (Tetric plus Fill) compared with a conventional composite (Filtek Supreme Ultra A2B). All RBCs were photocured using 3 s of extra-high-irradiance and using 10 s of high-irradiance exposure protocols. The empirical criterion for sufficient DoC was a bottom-to-top hardness ratio of at least 80% [9,36,39,40,41]. All the RBCs tested met their respective manufacturer claims for DoC. The results show RBC- and shade-dependent differences in curing characteristics and highlight the importance of selecting appropriate materials and light-curing protocols to achieve adequate polymerization and the clinical success of RBC restorations.

The first hypothesis was rejected. Tetric plus Fill Bleach plus, Tetric plus Fill A2 plus, and Tetric plus Fill A3 plus reached the 4 mm DoC with both exposure modes and Tetric plus Fill A3.5 reached 4 mm using the 10 s high exposure mode. These results were in accordance with the manufacturer’s instructions for use. The second hypothesis was rejected because the same DoC values were obtained with the 3 s and 10 s exposures for Tetric plus Fill Bleach, A2 and A3 and Filtek Supreme Ultra A2B, whereas different maximum depths were obtained for Tetric plus Fill A3.5. However, these results also met the manufacturer’s claimed DoC values.

The surface microhardness values were consistently higher for Filtek Supreme Ultra A2B than for Tetric plus Fill which is attributed to the higher filler content for the Filtek RBC compared to the Tetric RBCs. The higher surface hardness did not translate into a greater DoC. Filtek Supreme Ultra reached the 80% threshold to 2.0 mm with both exposure modes; therefore, the third hypothesis was rejected because Filtek Supreme Ultra A2B reached the manufacturer-recommended 2 mm increment thickness even when the 3 s exposure was used under the conditions of this study.

Figure 5 shows that Tetric plus Fill Bleach shade produced the greatest DoC, whereas the A3 plus and A3.5 plus shades produced lower DoC values. These findings show why the DoC should be reported separately for each shade and exposure protocol rather than inferred from a single bulk-fill claim. The response should not be attributed solely to radiant exposure. Material-specific photoinitiator chemistry and its optical transparency to light may also have contributed to the different curing profiles: Tetric plus Fill contains both camphorquinone and Ivocerin, whereas Filtek Supreme Ultra A2B uses camphorquinone as the reported photoinitiator. Therefore, differences in initiator absorption, efficiency, and polymerization kinetics should also be considered when interpreting the response of these composites to short, high-irradiance exposures.

These findings are consistent with previous studies showing that the DoC of bulk-fill RBCs is material-dependent and affected by specimen geometry, light delivery, and the method used to define the endpoint. The systematic review by Lima et al. reported that bulk-fill composites generally achieve greater DoC than conventional RBCs, but also highlighted the heterogeneity among materials and testing methods [7]. Flury et al. questioned whether the ISO 4049 scraping method is suitable for bulk-fill materials [34], and Alrahlah et al. reported material-dependent post-cure DoC values for bulk-fill composites [36]. Other studies have shown that the curing light, exposure time, specimen preparation, irradiation distance, and off-label short exposures can alter hardness and/or conversion at depth [31,33,51,54]. Thus, the present results should be interpreted as specific to the tested shades, LCU, exposure modes, and specimen geometry, not as a general ranking of all bulk-fill RBCs.

The conventional RBC, Filtek Supreme Ultra, had a steeper reduction in hardness with increasing depth. Although its surface HV values were higher than those of Tetric plus Fill, this observation was not the focus of the study and should not be interpreted as a formal between-material statistical comparison. The important finding is that the higher surface hardness and the higher irradiance used in the 3 s exposure did not produce a deeper DoC. Filtek Supreme Ultra reached the 80% threshold to 2.0 mm with both exposure modes. This result is consistent with the manufacturer’s recommended 2 mm maximum increment thickness [46]. The more pronounced drop in hardness with increasing depth observed in Filtek Supreme Ultra compared with Tetric plus Fill suggests greater light attenuation or less efficient polymerization kinetics at deeper layers for this material.

Clinically, the results support the general principle that dentists should follow the manufacturer’s instructions for use and should recognize that shade, material formulation, exposure mode, and increment thickness can all influence DoC. When access, light positioning, or shade/translucency is less favorable than in this controlled in vitro study, a longer exposure or thinner increment may be prudent.

Protecting the RBCs from light below 500 nm minimized this potential confounding factor that could affect polymerization of the RBC. Because it took over one hour to make all hardness measurements, immediate hardness testing was impractical. Storing the samples in darkness for 24 h in air at 37 °C before testing allowed most of the post-irradiation polymerization reaction to have finished [48] and provided stable hardness values that did not change while the RBCs were measured. The characterization of the LCU output, including spectral radiant flux and homogeneity, ensured that the light delivery was standardized and known, enabling this study to be replicated by others. Vickers microhardness testing at various depths proved to be a valid approach for evaluating DoC and supports its use by others [9,34,35,36,37,38]. The results were consistent with both manufacturers’ specifications [42,46]. The 300 g load [52,54,55,56,57] produced accurate and reproducible measurements without damaging the samples, as indicated by the preliminary load testing results, where higher loads of 2 kg caused damage to the specimens. The use of half-cylindrical molds that were filled with RBC and then assembled into a cylinder with each side separated by a 25 µm thick polyester strip was validated as a suitable mold configuration that allowed for consistent sample geometry. This polyester strip is thinner than most dental polyester strips, which are typically 50 µm thick.

A limitation of this study is that it is an in vitro evaluation, and the clinical relevance of the 80% bottom-to-top ratio should be considered in the broader context of material science and clinical performance standards [59]. The 80% ratio should not be interpreted as a precise biological threshold; however, values below this ratio suggest reduced polymerization at depth and may be associated with compromised mechanical properties, reduced elastic modulus, and increased release of residual monomers. The complex environment of the oral cavity, including the presence of saliva, temperature fluctuations, and occlusal forces, could potentially influence the long-term behavior of the cured RBCs. Only one Bluephase PowerCure LCU was used, although its output was characterized, and only one specimen geometry and storage condition were evaluated. Therefore, the results should not be generalized to all LCUs, all possible beam profiles, all cavity configurations, or all clinical conditions. Another limitation is that direct degree-of-conversion measurements were not made; Vickers microhardness was used as an indirect surrogate for polymerization at depth. While widely accepted [9,36,39,40,41], other criteria do exist [6,9,34,59], and properties such as the degree of conversion or the elastic modulus of the RBC may be adversely affected by rapid photocuring at a high irradiance [60]. In addition, because a 6 mm mold was used in the present study, these DoC values should be compared cautiously with ISO 4049 values obtained using a 4 mm mold and a scraping method.

## 5. Conclusions

Based on the 80% bottom-to-top Vickers microhardness ratio obtained after 24 h, the following conclusions can be drawn:

1. The simplified bulk-fill universal composite, Tetric plus Fill, met the manufacturer’s DoC claims.
2. Tetric plus Fill Bleach, A2 and A3 plus Fill reached their claimed DoC of 4.0 mm with both the 3 s extra-high and 10 s high exposure modes from the Bluephase PowerCure.
3. Tetric plus Fill A3.5 plus reached its claimed DoC of 3.5 mm with the extra-high 3 s exposure and 4.0 mm DoC with the 10 s high exposure from the Bluephase PowerCure.
4. The conventional composite, Filtek Supreme Ultra, achieved the 80% threshold to a depth of 2.0 mm using both the 3 s extra-high and 10 s high exposure modes from the Bluephase PowerCure.

## Figures and Tables

**Table 1:** Composition of the resin-based composites as provided by the manufacturers.

Product, Shade and Manufacturer	Abbreviation Used and Lot Number	Resin Matrix	Filler	Filler (wt. %/vol. %)
Tetric plus Fill: A2 plus. Ivoclar, Schaan, Liechtenstein	TpF A2 plus Cavifils (YM0205)	UDMA, Bis-GMA, aromatic-aliphatic UDMA, Bis-EMA, DCDMA, Tricyclodecane dimethanol dimethacrylate TCD-DMDA, Camphorquinone, Ivocerin.	Strontium glass, copolymer, mixed oxide (SiO_2_/ZrO_2_), ytterbium trifluoride	Total content of inorganic fillers: 70 wt%/51–52 vol% Particle size of the inorganic fillers: 0.01–3.0 μm
Tetric plus Fill: Bleach plus. Ivoclar, Schaan, Liechtenstein	TpF Bleach plus Cavifils (YM0208)	UDMA, Bis-GMA, aromatic-aliphatic UDMA, Bis-EMA, DCDMA, Tricyclodecane dimethanol dimethacrylate TCD-DMDA, Camphorquinone, Ivocerin.	Strontium glass, copolymer, mixed oxide (SiO_2_/ZrO_2_), ytterbium trifluoride	Total content of inorganic fillers: 70 wt%/51–52 vol% Particle size of the inorganic fillers: 0.01–3.0 μm
Tetric plus Fill: A3 plus. Ivoclar, Schaan, Liechtenstein	TpF A3 plus Cavifils (YM0206)	UDMA, Bis-GMA, aromatic-aliphatic UDMA, Bis-EMA, DCDMA, Tricyclodecane dimethanol dimethacrylate TCD-DMDA, Camphorquinone, Ivocerin.	Strontium glass, copolymer, mixed oxide (SiO_2_/ZrO_2_), ytterbium trifluoride	Total content of inorganic fillers: 70 wt%/51–52 vol% Particle size of the inorganic fillers: 0.01–3.0 μm
Tetric plus Fill: A3.5 plus. Ivoclar, Schaan, Liechtenstein	TpF A3.5 plus Cavifils (YM0207)	UDMA, Bis-GMA, aromatic-aliphatic UDMA, Bis-EMA, DCDMA, Tricyclodecane dimethanol dimethacrylate TCD-DMDA, Camphorquinone, Ivocerin.	Strontium glass, copolymer, mixed oxide (SiO_2_/ZrO_2_), ytterbium trifluoride	Total content of inorganic fillers: 70 wt%/51–52 vol% Particle size of the inorganic fillers: 0.01–3.0 μm
3M™ Filtek™ Supreme Ultra Universal Restorative: A2B (Body). Solventum (formerly 3M Oral Care), St. Paul, MN, USA	FSU A2B	Bis-GMA; Bis-EMA(6); UDMA; minor TEGDMA and PEGDMA (viscosity/shrinkage modifiers) Camphorquinone	Silane-treated zirconia and silica. Non-agglomerated/non-aggregated 20 nm silica; non-agglomerated/non-aggregated 4–11 nm zirconia; aggregated zirconia/silica nanoclusters (≈0.6–10 µm clusters for dentin/enamel/body shades).	Inorganic nanofiller filler loading (A2B body shade): ~78.5 wt%/63.3 vol%.

**Bis-GMA:** bisphenol A-glycidyl methacrylate. **Bis-EMA:** ethoxylated bisphenol A dimethacrylate. **BIS-EMA-6:** ethoxylated bisphenol A dimethacrylate (average ~6 ethoxy repeat units). **UDMA:** urethane dimethacrylate. **DCDMA:** dicycloalkyl dimethacrylate monomer. **TCD-DMDA:** tricyclodecane dimethanol dimethacrylate. **PEGDMA:** polyethylene glycol dimethacrylate. **TEGDMA:** triethylene glycol dimethacrylate. **EDMAB:** ethyl p-dimethylaminobenzoate.

**Table 2:** Mean Vickers microhardness (HV) ± standard error (SE) at 0.5 mm depth intervals for the five composites (RBCs), with separate rows for each exposure protocol (3 s on the extra-high irradiance mode or the 10 s high irradiance mode).

RBC	Exposure Protocol	HV 0.0 mm	HV 0.5 mm	HV 1.0 mm	HV 1.5 mm	HV 2.0 mm	HV 2.5 mm	HV 3.0 mm	HV 3.5 mm	HV 4.0 mm	HV 4.5 mm	HV 5.0 mm
**TpF A2 plus**	3 s	63.16 (0.61)	63.22 (1.46)	61.05 (1.15)	59.49 (0.94)	57.92 (0.97)	57.32 (0.63)	55.76 (0.63)	53.01 (0.50)	47.34 (0.89)	39.90 (1.19)	31.97 (0.97)
	10 s	59.21 (1.27)	60.53 (1.11)	62.98 (0.80)	62.82 (0.52)	62.20 (0.35)	58.58 (0.66)	57.48 (0.55)	55.35 (0.42)	51.05 (0.63)	45.20 (0.89)	36.70 (1.07)
**TpF A3 plus**	3 s	63.56 (0.42)	60.23 (1.00)	60.58 (1.17)	61.41 (0.53)	59.50 (0.92)	57.79 (0.76)	56.16 (0.91)	53.61 (0.84)	49.76 (0.87)	44.21 (1.13)	35.92 (1.64)
	10 s	62.95 (0.89)	64.62 (2.54)	61.29 (0.87)	60.00 (1.06)	59.95 (0.95)	58.81 (0.44)	55.51 (0.87)	52.92 (0.80)	49.94 (0.75)	44.56 (0.93)	37.54 (1.11)
**TpF A3.5 plus**	3 s	61.85 (1.13)	62.13 (0.36)	62.38 (0.71)	60.61 (0.57)	58.88 (0.60)	56.34 (0.60)	53.70 (0.62)	50.36 (0.87)	45.40 (0.74)	38.11 (0.71)	28.09 (0.94)
	10 s	62.43 (1.04)	62.24 (1.25)	61.25 (0.99)	58.79 (0.75)	60.29 (0.66)	57.43 (0.80)	54.56 (0.91)	49.59 (1.11)	47.19 (0.89)	39.51 (1.02)	31.68 (1.21)
**TpF Bleach plus**	3 s	62.32 (0.68)	58.00 (1.47)	61.72 (0.81)	62.51 (0.46)	60.09 (0.68)	58.77 (0.75)	58.10 (0.51)	55.46 (0.65)	52.22 (0.83)	47.60 (0.99)	39.63 (1.42)
	10 s	60.54 (1.10)	62.29 (1.11)	62.81 (1.25)	62.88 (0.65)	61.12 (0.94)	61.66 (0.36)	59.20 (0.50)	57.11 (0.66)	54.16 (0.62)	51.02 (0.74)	45.99 (0.80)
**FSU A2B**	3 s	95.78 (0.94)	96.77 (0.90)	94.71 (0.84)	90.64 (0.56)	83.64 (0.92)	73.19 (1.26)	57.37 (1.68)	37.32 (1.90)	21.71 (1.35)	TOO SOFT	TOO SOFT
	10 s	98.44 (0.92)	91.93 (2.08)	93.98 (0.65)	91.98 (0.61)	86.83 (0.84)	76.82 (1.19)	65.16 (1.43)	48.15 (1.79)	30.31 (1.82)	TOO SOFT	TOO SOFT

## Data Availability

The original contributions presented in this study are included in the article. Further inquiries can be directed to the corresponding author.
